# Impedimetric Sensing of Factor V Leiden Mutation by Zip Nucleic Acid Probe and Electrochemical Array

**DOI:** 10.3390/bios10090116

**Published:** 2020-09-07

**Authors:** Arzum Erdem, Ece Eksin

**Affiliations:** Analytical Chemistry Department, Faculty of Pharmacy, Ege University, Bornova, Izmir 35100, Turkey; eceksin@hotmail.com

**Keywords:** 8-channel screen-printed electrochemical arrays, zip nucleic acids, SNP, electrochemical impedance spectroscopy

## Abstract

A carbon nanofiber enriched 8-channel screen-printed electrochemical array was used for the impedimetric detection of SNP related to Factor V Leiden (FV Leiden) mutation, which is the most common inherited form of thrombophilia. FV Leiden mutation sensing was carried out in three steps: solution-phase nucleic acid hybridization between zip nucleic acid probe (Z-probe) and mutant type DNA target, followed by the immobilization of the hybrid on the working electrode area of array, and measurement by electrochemical impedance spectroscopy (EIS). The selectivity of the assay was tested against mutation-free DNA sequences and synthetic polymerase chain reaction (PCR) samples. The developed biosensor was a trustful assay for FV Leiden mutation diagnosis, which can effectively discriminate wild type and mutant type even in PCR samples.

## 1. Introduction

Laboratory analyzers are suited for a hospital setting and requires trained personnel to operate the analyzer and interpret the results accurately. However, biosensors have many advantages since they are user-friendly and provide results rapidly with high sensitivity. A great demand for accurate monitoring of biomarkers related to important diseases is the driving force toward the development of novel analytical tools for diagnostics [1,2].

Many researchers are interested in the development of fast screening tools for clinical use. These tools are mostly based on optical or spectroscopic techniques. However, electrochemical methods have many strong points with regard to other techniques such as simple instrumentation, easy to use, low cost, and low sample necessity [3]. Electrochemical biosensors provide direct analysis of various analytes within minutes. Therefore, electrochemical biosensors can be considered as the most appropriate tool for clinical diagnosis by means of their superior features as above-mentioned. Electrochemical DNA biosensor arrays, as a member of the electrochemical biosensor family, are widely used as powerful tools for the diagnosis of genetic and infectious diseases [4,5]. Many recent works have demonstrated the usage of screen-printed electrode arrays in order to carry out multiple measurements at the same time [5,6,7,8,9]. Recently, our group developed sensitive biosensors for the detection of protein, microRNA, and SNPs [10,11,12,13,14,15].

FV Leiden, with the most common inherited prothrombotic conditions, occurs due to a single point mutation of the coagulation factor V gene in the chromosome [16]. Between 3% and 8% of Europeans carry the one copy of the Factor V Leiden mutation, and about 1 in 5000 people have two copies of the FV Leiden mutation. Inheriting one copy slightly increases the risk of developing blood clots. Furthermore, inheriting two copies—one from each parent—significantly increases the risk of developing blood clots. These abnormal clots can lead to long-term health problems or become life-threatening [17]. Due to the importance of FV Leiden mutation sensing, there is an urgent need to develop sensitive, reliable, and fast detection protocols for the FV Leiden mutation. Under this scope, the quantitative analysis of Factor V Leiden was carried out by different methodologies such as the immunosorbent assay [18], fluorescent assay [19], and sandwich-optical sensing method [20]. However, these methods require expensive facilities and complex procedures in combination with the use of radioactive/fluorescent tags. Therefore, these methods are not suitable for the development of simple and low-cost point-of-care (PoC) devices.

Zip nucleic acids contain a cationic compound, spermine, that has an impact on the affinity between the oligonucleotide and its target nucleic acid. Therefore, single base-mismatched sequences or single nucleotide polymorphisms (SNPs) can be successfully discriminated by using zip nucleic acid probes (Z-probes) [21]. Recent studies have confirmed the reliability of Z-probes such as primers, real-time PCR probes, and splice switching oligonucleotides (SSOs) [22,23,24,25]. Z-probe applications also include miRNA detection, detection of AT-rich sequences, in situ hybridization, etc. [26,27]. Furthermore, Z-probes were used as an efficient probe for the development of the impedimetric detection protocol of single nucleotide mutation related to FV Leiden in our previous study [28]. Before and after solution-phase hybridization occurred between Z-probes and its mutant type DNA target, the impedimetric measurement was performed by carbon nanofiber enriched screen-printed electrodes. The impedimetric detection of different single point mutations such as G to A, G to C, and G to T in short DNA oligonucleotides was successfully carried out. The discrimination between mutant type DNA (G to A) and wild type DNA was explored successfully even though the target sequence with a mutation (G to A) was at the 3‘-end position of both PCR products at the length of 143 nt or 220 nt. In addition, the detection of any other SNPs (G to C, or G to T) was performed in solution phase hybridization more selectively by using Z-probes in contrast to the DNA probe. Please note that this is a follow-up study of our previous study.

Herein, EIS based sensing protocol for of the FV Leiden mutation was performed by 8-channel screen-printed electrochemical arrays. In order to improve the efficiency of solution-phase nucleic acid hybridization between Z-probes and target sequence, the experimental parameters were optimized. The selectivity of the assay was tested against wild type DNA sequence and synthetic PCR samples.

## 2. Materials and Methods

### 2.1. Instruments and Chemicals

AUTOLAB-302 PGSTAT with the GPES 4.9.007 software package (Eco Chemie, Utrecht, The Netherlands) was used for electrochemical impedance (EIS) measurements.

The detailed information about oligonucleotides, PCR products, and carbon nanofibers enriched 8-channel screen-printed electrochemical arrays can be found in the Supplementary Materials.

### 2.2. Methodology

The following steps were carried out for EIS-based sensing of the FV Leiden mutation.

(i). Hybridization of the Z-probe with mutant type DNA, or wild type DNA, or C-mutant type DNA, T-mutant type DNA, ODN-1, ODN-2, mutant type, and wild type PCR in the solution phase;
(ii). Immobilization of the hybridization products on the working electrode area of the array electrode;
(iii). Impedimetric measurements.

The desired concentrations of the Z-probe (or DNA probe) and mutant type DNA (or any other of oligonucleotides (ODNs)) were prepared in phosphate buffer saline (PBS, pH, 7.4) and mixed in the ratio of 1:1 (v:v). This mixture was allowed to undergo solution-phase hybridization for 10 min with gentle mixing at 400 rpm under room temperature using a Thermo-Shaker (Biosan, Latvia).

**Scheme 1:**
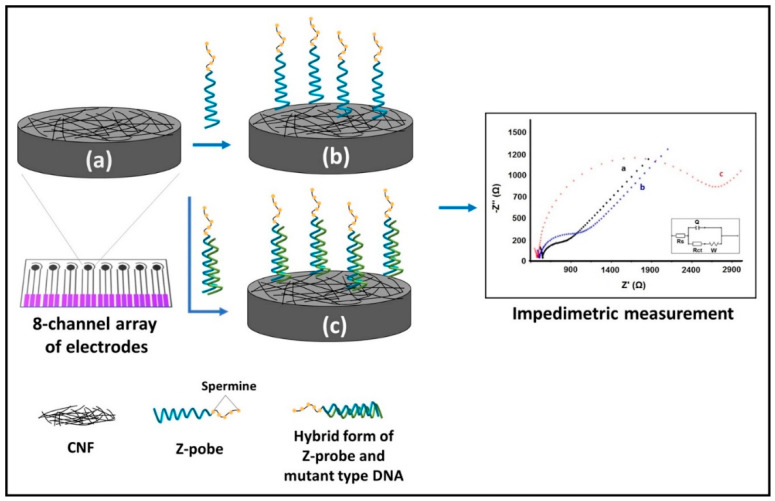
The representative experimental scheme related to electrochemical impedance spectroscopy based sensing of the FV Leiden mutation. (**a**) Electrode control, (**b**) Z-probe in the absence of target, (**c**) the hybrid form of Z-probe and mutant type DNA target.

The solution containing hybrids of the Z-probe-DNA target or DNA probe-DNA target was dropped onto the surface of the working electrode of the array system and incubated for 15 min. Immobilization of the hybrids was performed according to the drop-casting method. After that, the electrodes were washed with PBS before measurement. The representative scheme is given in Scheme 1.

### 2.3. Electrochemical Impedance Spectroscopy Measurements

EIS measurements were performed as reported in our previous work [28]. The Randles circuit was used as the equivalent circuit model used for fitting EIS data, which is shown as the inset in all Nyquist diagrams.

## 3. Results

We aimed to apply our previous assay [28] to the 8-channel array of electrodes in order to perform multiple simultaneous analysis. This array of electrodes has been successfully employed in numerous applications based on electrochemical biosensors [10,11,12,13,14,15,29,30] while presenting their great advantages such as low sample requirement and easy implementation to point of care (PoC) system. The characterization of the carbon nanofiber enriched electrodes has been given in earlier reports [28,31,32,33].

**Figure 1:**
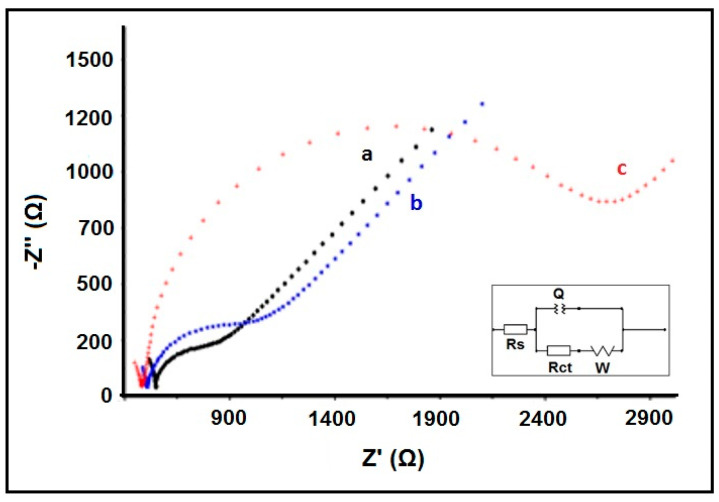
The Nyquist diagrams obtained after the hybridization of the Z-probe and mutant type DNA target under optimized conditions. (**a**) Electrode control, (**b**) Z-probe in the absence of target, (**c**) the hybrid form of Z-probe and mutant type DNA target.

The impedimetric sensing performance and operational characteristics of the array biosensor were evaluated after optimizing the experimental conditions (Figures S1–S7). The optimized variables are given in Table 1 and the Nyquist diagrams related to the hybridization of the Z-probe and mutant type DNA target under optimized conditions are given in Figure 1.

**Figure 2:**
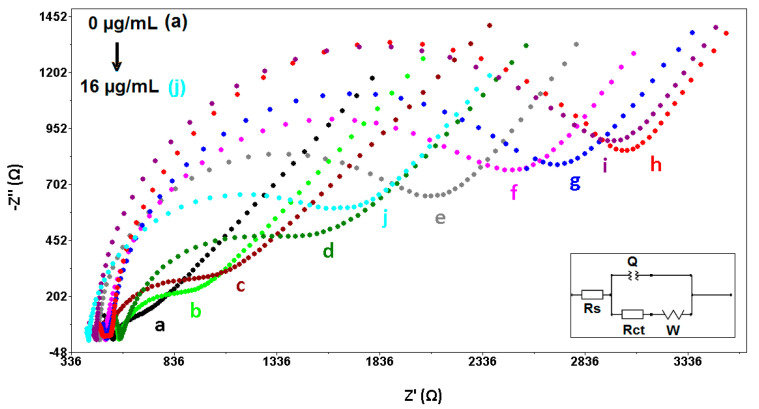
Nyquist diagrams related to (**a**) electrode control, (**b**) 1.0 µg mL^−1^ Z-probe in the absence of target, the hybrid form of Z-probe and (**c**) 2.0, (**d**) 4.0, (**e**) 6.0, (**f)** 8.0, (**g**) 10.0, (**h**) 12.0, (**i**) 14.0, (**j**) 16.0 µg mL^−1^ mutant type DNA target.

The effect of mutant type DNA target concentration on the hybridization process was investigated. Figure S8 shows the line graph for the tested mutant type DNA target concentrations. There was an increase at R_ct_ up to 12.0 µg mL^−1^, then a decrease at R_ct_ was recorded up to 16.0 µg mL^−1^. As shown in Figure S8, the highest R_ct_ value was measured at 12.0 µg mL^−1^ in mutant type DNA target of 3226.0 ± 456.6 Ω with the RSD%, 14.2% (n = 3); the upper limit of the linear range was chosen as 12.0 µg mL^−1^. A linearity in the response based on the R_ct_ value was obtained in the mutant type DNA concentration range varying from 2.0 to 10.0 µg mL^−1^. The representative Nyquist diagrams are shown in Figure 2. The limit of detection was estimated by using the Miller and Miller technique [34] and was found to be 1.9 µg mL^−1^ (equal to 266.0 nM, 2.6 pmol in the 10.0 µL sample) according to the calibration plot shown in the Figure S8 inset with the equation of y = 208.96x + 235.66. In addition, the sensitivity was calculated and found to be 2149.8 Ω·mL/µg·cm^2^.

The selectivity of the Z-probe to mutant type DNA target was then tested against the wild type DNA target. The same experiment was performed with the DNA probe instead of the Z-probe in order to compare the performance of the Z-probe.

**Figure 3:**
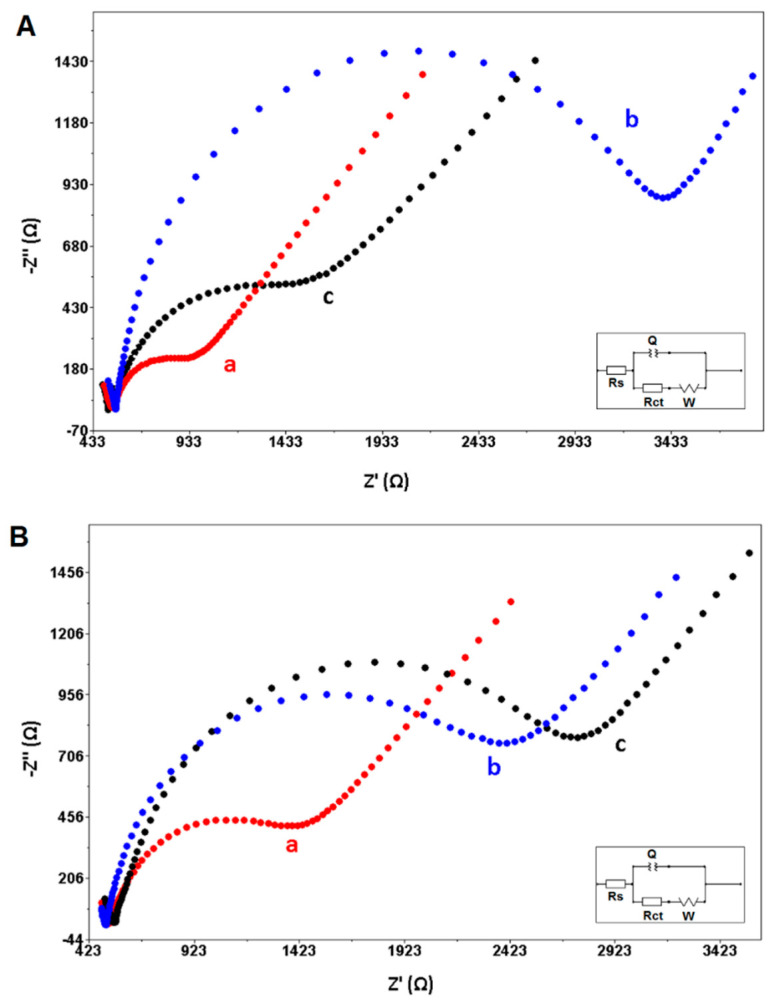
The hybridization between 1.0 µg mL^−1^ (**A**) Z-probe, or (**B**) DNA probe and 12.0 µg mL^−1^. mutant type DNA or wild type DNA target. Nyquist diagrams related to (a) Z-probe or DNA probe in the absence of target, the hybrid form of Z-probe, or DNA probe with (b) mutant type DNA target, (c) wild type DNA target.

The average R_ct_ value was obtained of 3219.0 ± 373.0 Ω (RSD%, 11.6%, n = 3) after hybridization of the Z-probe and mutant type DNA target (Figure 3A). However, it was 1083.0 ± 65.0 Ω (RSD%, 6.0%, n = 3) after the hybridization of the Z-probe with the wild type DNA target. On the other hand, the average R_ct_ value was obtained of 2504.0 ± 629.3 Ω (RSD%, 25.1%, n = 3) in the presence of hybrid of the DNA probe and mutant type DNA target (Figure 3B), and 2327.0 ± 89.1 Ω (RSD%, 3.8%, n = 3) with the hybrid of the DNA probe and wild type DNA target. According to the efficiency of hybridization% (H_Eff_%), it can be said that the Z-probe exhibited a selective behavior to its wild type DNA target (shown in Table 2). Nevertheless, the DNA probe was not selective enough to the wild type target.

The selectivity of the impedimetric assay based on the Z-probe and nanomaterials enriched array biosensor was tested over different oligonucleotides with different single-base mutations located at mutant type DNA target sequence, or noncomplementary sequences, C-mutant type DNA or T-mutant type DNA or ODN-1 or ODN-2 (Figures S9 and S10). The average R_ct_ with the H_Eff_% is given in Table S1. The highest H_Eff_% was found to be 89% with the hybrid form of the Z-probe and mutant type DNA. A selective behavior of the Z-probe was monitored even in the presence of DNA oligonucleotides with different single-base mutation or noncomplementary ODNs.

The impedimetric sensing of FV Leiden mutation in the PCR products with the length of 143 nt was analyzed using the Z-probe accordingly, and presented comparatively with the DNA probe. The hybridization of the 1.0 µg mL^−1^ Z-probe and 12.0 µg mL^−1^ (equals to 0.3 µM) mutant type PCR, or wild type PCR was performed under optimum experimental conditions and the average R_ct_ with H_Eff_% values is shown in Table 3.

The H_Eff_% value obtained by the Z-probe (86.0%) was found to be higher than the one obtained by the DNA probe (53.0%). Hence, the discrimination of a single-base mutation was selectively and sensitively explored in the presence of the Z-probe, even when the target sequence was part of the PCR product with the length of 143 nt.

The earlier studies related to the detection of DNA using the array of electrodes are listed in Table 4 and compared to the present study.

## 4. Conclusions

The impedimetric analysis of the FV Leiden mutation was carried out by 8-channel screen-printed electrochemical arrays in a relatively shorter time (i.e., 30 min) compared to previous works performed by different arrays of electrodes [13,14,15,35,36,37,38,39,40,41,42,43,44,45,46,47] (see Table 4). Using screen-printed electrochemical arrays, a single-base mutation successfully discriminated complementary target DNAs by means of the Z-probe. The LODs were calculated and found to be 1.9 µg mL^−1^ (266.0 nM). Based on our experiences, the Z-probe based impedimetric biosensors will be at the front of furthering the expansion of a new generation of nucleic acids on the development of PoC devices.

## Figures and Tables

**Table 1:** Experimental variables optimized for the impedimetric sensing performance of the array biosensor.

Variable	Evaluated Conditions	Optimum Condition
Hybridization temperature, °C	25, 50, 75	25
Hybridization buffer, pH	ABS (pH, 4.8) PBS (pH, 7.4) CBS (pH, 9.5)	PBS (pH 7.4)
[Mg^2+^] in hybridization buffer, mM	NA *, 0.5, 1	NA *
Hybridization time, minute	5, 10, 15	10

* NA: There is no Mg^2+^ available in hybridization buffer.

**Table 2:** The average R_ct_ values measured with the hybrid form of the Z-probe or DNA probe with mutant type DNA target/wild type DNA target and H_Eff_% values.

	R_ct_ (Ω)	H_Eff_%
Z-probe	369.4 ± 59.9	-
Z-probe and mutant type DNA target	3219.0 ± 373.0	89.0
Z-probe and wild type DNA target	1083.0 ± 65.0	65.0
DNA probe	848.0 ± 209.0	-
DNA probe and mutant type DNA Target	2504.0 ± 629.3	66.0
DNA probe and wild type DNA target	2327.0 ± 89.1	63.0

**Table 3:** The average R_ct_ values measured with the hybrid form of the Z-probe or DNA probe with mutant type PCR or wild type PCR with the values of H_Eff_%.

	R_ct_ (Ω)	H_Eff_%
Z-probe	369.4 ± 59.9	-
Z-probe and mutant type PCR	2608.0 ± 361.3	86.0
Z-probe and wild type PCR	1923.3 ± 516.4	81.0
DNA probe	848.0 ± 209.0	-
DNA probe and mutant type PCR	1795.0 ± 481.2	53.0
DNA probe and wild type PCR	1848.5 ± 379.7	54.0

**Table 4:** The earlier studies developed for the detection of DNA by the array of electrodes in contrast to the present study.

Electrode	Modification	Analite	Technique	Assay Time	LOD	Reference
8-channel array of electrodes	Carbon nanofiber	DNA (FV Leiden mutation)	DPV	135 min	1.6 µM	[13]
8-channel array of electrodes	Carbon nanofiber	DNA (FV Leiden mutation)	DPV	35 min	0.4 µM	[14]
8-channel array of electrodes	Carbon nanofiber	DNA (FV Leiden mutation)	EIS	50 min	133.0 nM	[15]
Gold film electrode array chip	-	DNA	SWV	NA	NA	[35]
Three-dimensional interdigitated electrode array	Silane	DNA	EIS	24 h	NA	[36]
ITO electrode array	Graphene-mesoporous silica hybrid nanosheets	DNA	DPV	70 min	10.0 fM	[37]
nanodisk-array electrodes	polystyrene-block-poly(methylmethacrylate)-derived thin films	DNA	CV	2 h	0.4 nM–4.2 nM	[38]
32 microelectrode Chips (gold)	-	DNA	CV	30 min		[39]
ITO electrode array on glass wafer	-	H1N1 influenza virus DNA	Capacitance	3.5 h	3.9 nM	[40]
16 thru-hole array on printed circuit board	-	Hepatitis A-B-C virus DNA	ECL	3.5 h	NA	[41]
Gold electrode microarray	-	DNA	EIS	22 h	1.0 pM	[42]
16-gold electrode sensor arrays	-	DNA	CV, Amperometry	3 h	NA	[43]
electrode array housed within the microfluidic cell	-	*Karlodinium armiger* DNA	SWV, Chronoamperometry	17 h	277.0 aM	[44]
120-channel gold microelectrode array chip	-	miRNA	CV	1 h	140.0 zmol	[45]
multi-electrode array (6-gold electrode)	-	HIV-1, HIV-2 DNA	SWV	7.5 h	0.1 nM	[46]
16 microwells- column electrode	-	DNA	Amperometry	2 h	30.0 nM	[47]
8-channel array of electrodes	Carbon nanofiber	DNA (FV Leiden mutation)	EIS	25 min	266.0 nM	Present work

## Supplementary Materials

The following are available online at https://www.mdpi.com/2079-6374/10/9/116/s1. Figure S1. The Nyquist diagrams obtained after the hybridization of 2.0 µg mL^−1^ (A) DNA probe, (B) 3’Z-probe, (C) 5’Z-probe and 10.0 µg mL^−1^ mutant type DNA target at 25 °C. (a) electrode itself, (b) the pseudo-hybridization of DNA probe, 3’Z-probe or 5’Z-probe, (c) the hybridization of DNA probe, 3’Z-probe or 5’Z-probe and mutant type DNA target. (D) Histograms representing the average R_ct_ values obtained by (a) electrode itself, the pseudo-hybridization of 2.0 µg mL^−1^ (b) DNA probe, (d) 3’Z-probe or (f) 5’Z-probe, the hybridization between 2.0 µg mL^−1^ (c) DNA probe, (e) 3’Z-probe or (g) 5’Z-probe and 10.0 µg mL^−1^ mutant type DNA target. Figure S2. Nyquist diagrams obtained by (a) electrode itself, the immobilization of 2.0 µg mL^−1^ of (b) spermine or (c) Z-probe, and the interaction/hybridization of 2.0 µg mL^−1^ of (d) spermine or (e) Z-probe with 10.0 µg mL^−1^ mutant type DNA target. Figure S3. The Nyquist diagrams obtained after the hybridization of 2.0 µg mL^−1^ Z-probe and 10.0 µg mL^−1^ mutant type DNA target at (A) 25 °C, (B) 50 °C and (C) 75 °C. (a) electrode itself, (b) the pseudo-hybridization of Z-probe at 25 °C, 50 °C and 75 °C, (c) the hybridization of Z-probe and mutant type DNA target at 25 °C, 50 °C and 75 °C. Figure S4. The Nyquist diagrams obtained after the hybridization of 2 µg mL^−1^ Z-probe and 10.0 µg mL^−1^ mutant type DNA target in (A) ABS (pH 4.8), (B) PBS (pH 7.4) and (C) CBS (pH 9.5). (a) electrode itself, (b) the pseudo-hybridization of Z-probe in ABS (pH 4.8), PBS (pH 7.4) or CBS (pH 9.5). (c) the hybridization of Z-probe and mutant type DNA target in ABS (pH 4.8), PBS (pH 7.4) or CBS (pH 9.5). Inset was the equivalent circuit model used for fitting of the impedance datas. Figure S5. The Nyquist diagrams obtained after the hybridization of 2.0 µg mL^−1^ Z-probe and 10.0 µg mL^−1^ mutant type DNA target in (A) PBS (pH 7.4) or (B) 0.5 mM and (C) 1.0 mM Mg^2+^ contained PBS (pH 7.4). (a) electrode itself, (b) the pseudo-hybridization of Z-probe in PBS (pH 7.4), or PBS (pH 7.4) containing 0.5 mM or 1.0 mM Mg^2+^. (c) the hybridization of Z-probe and mutant type DNA target in PBS (pH 7.4), or PBS (pH 7.4) containing 0.5 mM or 1.0 mM Mg^2+^. Figure S6. The Nyquist diagrams obtained after the hybridization of 2.0 µg mL^−1^ Z-probe and 10.0 µg mL^−1^ mutant type DNA target during 5 min, 10 min and 15 min. (a) electrode itself, the pseudo-hybridization of Z-probe during (b) 5 min, (c) 10 min, (d) 15min, the hybridization of Z-probe and mutant type DNA target during (b’) 5 min, (c’) 10 min and (d’) 15 min. Figure S7. Nyquist diagrams of (a) electrode itself, before (A) and after (B) the hybridization of (b) 0.25, (c) 0.5, (d) 1.0, (e) 2.0 and (f) 4.0 µg mL^−1^ Z-probe and 10.0 µg mL^−1^ mutant type DNA target. Figure S8. Line graph representing the R_ct_ values recorded by the hybridization of 1.0 µg mL^−1^ Z-probe and mutant type DNA target at the concentration level from 2.0 to 16.0 µg/mL. Inset: Calibration graph based on the average R_ct_ values (*n* = 3) obtained after the hybridization of Z-probe with mutant type DNA target in the concentration range from 2.0 to 10.0 µg mL^−1^. Figure S9. The hybridization of 1.0 µg mL^−1^ Z-probe and 12.0 µg mL^−1^ mutant type DNA target or C-mutant type DNA or T-mutant type DNA. (A) Nyquist diagrams, (B) histograms representing the R_ct_ values obtained by (a) electrode itself, (b) the pseudo-hybridization of Z-probe, after the hybridization of Z-probe and (c) mutant type DNA target, (d) C-mutant type DNA, (e) T-mutant type DNA. Figure S10. The hybridization of Z-probe and mutant type DNA target or ODN-1 or ODN-2. (A) Nyquist diagrams, (B) histograms representing the R_ct_ values obtained by (a) electrode itself, (b) presudo-hybridization of Z-probe, after the hybridization of Z-probe and (c) mutant type DNA target, (d) ODN-1, (e) ODN-2 (*n* = 3). Table S1. H_Eff_% calculated based on the average R_ct_ value obtained after the hybridization of Z-probe with mutant type DNA target/C-mutant type DNA/T-mutant type DNA/ODN-1/ODN-2 in contrast to the average R_ct_ value obtained in the presence of pseudo hybridization.
