# Supplementary material for: Impedimetric Sensing of Factor V Leiden Mutation by Zip Nucleic Acid Probe and Electrochemical Array
Source: Biosensors (Basel). 2020 Sep 7;10(9):116. doi: 10.3390/bios10090116 (PMC7559847; doi:10.3390/bios10090116)
Supplement: Supplementary file 1 [file biosensors-10-00116-s001.pdf]

Supplementary information

Impedimetric Sensing of Factor V Leiden Mutation by Zip Nucleic Acid Probe and Electrochemical Array

The base sequences of oligonucleotides and PCR products are listed below as given in our earlier reports [13-15,28]. Mutations shown in bold letters.

5′ Z-probe (Inosine substituted, 23 nt):

5′-5S-AAT ACC TIT ATT CCT TIC CTI TC-3′ (S: Spermine, I: Inosine)

3′ Z-probe (Inosine substituted, 23 nt):

5′- AAT ACC TIT ATT CCT TIC CTI TC-5S-3′ (S: Spermine, I: Inosine)

DNA probe (Inosine substituted, 23 nt):

5′-AAT ACC TIT ATT CCT TIC CTI TC-3′ (I: Inosine)

Mutant type DNA target (23 nt):

5′-GAC AGG C**A**A GGA ATA CAG GTA TT-3′

Wild type DNA (23 nt):

5′-GAC AGG C**G**A GGA ATA CAG GTA TT-3′

C- mutant type DNA oligonucleotide (23 nt):

5′-GAC AGG C**C**A GGA ATA CAG GTA TT-3′

T-mutant type DNA oligonucleotide (23 nt):

5′-GAC AGG C**T**A GGA ATA CAG GTA TT-3′

Non-complementary DNA oligonucleotide-1 (ODN-1, 20 nt):

5′-AAT ACC ACA TCA TCC ATA TA-3′

Non-complementary DNA oligonucleotide-2 (ODN-2, 23 nt):

5′-AAT ACC TGT ATT CCT CGC CTG TC-3′

Mutant type PCR (143 nt):

5′-ACC CAC AGA AAA TGA TGC CCA GTG CTT AAC AAG ACC ATA CTA CAG TGA CGT GGA CAT CAT GAG AGA CAT CGC CTC TGG GCT AAT AGG ACT ACT TCT AAT CTG TAA GAG CAG ATC CCT GGA CAG GC**A** AGG AAT ACA GGT ATT TT-3′

Wild type PCR (143 nt):

5'-ACC CAC AGA AAA TGA TGC CCA GTG CTT AAC AAG ACC ATA CTA CAG TGA CGT GGA CAT CAT GAG AGA CAT CGC CTC TGG GCT AAT AGG ACT ACT TCT AAT CTG TAA GAG CAG ATC CCT G<u>GA CAG GC**G** AGG AAT ACA GGT ATT TT</u>-3'

The Z-probe, DNA oligonucleotides and PCR products were purchased from (as lyophilized powder) TIB Molbiol (Germany).

Z-probe stock solution as 472.0 µg mL^{-1} was prepared in Dulbecco's modified Phosphate Buffer Solution (pH 7.4) and kept frozen. The stock solutions of other oligonucleotides were prepared in ultrapure water (i.e, RNase/DNase free). The diluted solutions of Z-probe, DNA probe, wild type DNA, mutant type DNA, C-mutant type DNA, T-mutant type DNA, ODN-1, ODN-2 and PCR products were prepared in 50 mM phosphate buffer solution containing 20 mM NaCl (PBS, pH 7.4). The diluted solutions of mutant type DNA target was prepared in PBS (pH 7.4). Other chemicals were in analytical reagent grade and they were supplied from Sigma-Aldrich and Merck.

Carbon nanofiber enriched 8-channel screen-printed electrochemical array

Graphitized carbon nanofibers enriched 8-channel screen-printed electrochemical arrays were purchased from DropSens (Oviedo-Asturias, Spain). A specific connector (µStat 8000/P Connector) purchased from DropSens (Oviedo-Asturias, Spain) that allows the connection of electrodes to the potentiostat.

After placing a 10 µL drop of the corresponding solution to the working area of these electrodes, each measurement was respectively performed as introduced in our earlier reports [13-15].

The hybridization efficiency (H_{Eff}%) was calculated for each hybridization occured between DNA probe/Z-probe with mutant type/wild type DNA, mutant type/wild type PCR according to the equation 1.

$$\textbf{The hybridization efficiency } (H_{Eff}\%) = [\Delta R_{ct} / R_{ct\ hybrid}] \times 100 \qquad \textbf{Eq 1.}$$

$$(\Delta Rct = R_{ct\ hybrid} - R_{ct\ probe})$$

The hybridization efficiency (H_{Eff}%) was calculated based on the results related to each experiment on possible hybridization between DNA probe/Z-probe with mutant type DNA/wild type DNA target or mutant type PCR/wild type PCR (Eq. 1). The higher H_{Eff}% value is expected in the case of hybridization of Z-probe with mutant type DNA/mutant type PCR comparison to the one with wild type DNA/wild type PCR. In addition, the higher H_{Eff}% is expected in case of hybridization between Z-probe and its target DNA in contrast to DNA probe. Therefore, we can consider that Z-probe can recognize SNP in more selectively than DNA probe.

The optimization of experimental parameters for detection of FV Leiden mutation

Firstly, DNA probe, 3'Z-probe and 5'Z-probe was compared by means of the nucleic acid hybridization efficiency and the results were shown in Figure S1. For this purpose, the hybridization of 2.0 µg mL^{-1} of

the probes and 10.0 μg mL^{-1} (equals to 1.4 μM) mutant type DNA target was performed and impedimetric measurements were done. After the pseudo hybridization of DNA probe, 3'Z-probe and 5'Z-probe, the average R_{ct} values were recorded as 1248.0 ± 113.1 Ω, 436.0 ± 134.4 Ω and 386.3 ± 47.9 Ω with the RSDs% as 9.1%, 30,8% and 12.4%, respectively. After the full match hybridization of DNA probe, 3'Z-probe or 5'Z-probe and mutant type DNA target, the average R_{ct} values 1.9, 3.8 and 5.7 fold increased and measured as 2469.5 ± 293.4 Ω, 1635.0 ± 43.8 Ω and 2207.4 ± 214.5 Ω with the RSDs% as 11.8%, 2.7% and 9.7% respectively. The highest increase at the R_{ct} value was obtained in the presence of 5'Z-probe. Thus, 5'Z-probe was used in our further experimental steps.

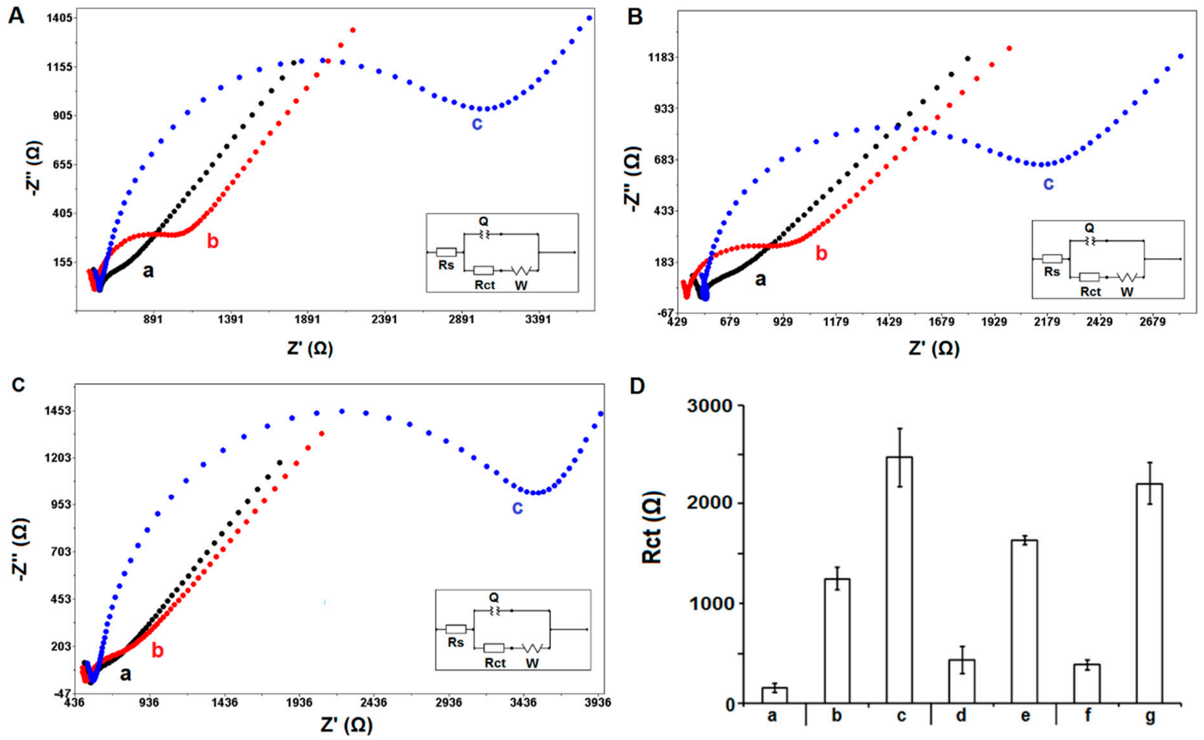

Figure 1. The Nyquist diagrams obtained after the hybridization of 2.0 μg mL^{-1} **(A)** DNA probe, **(B)** 3' Z- probe, **(C)** 5'Z-probe and 10.0 μg mL^{-1} mutant type DNA target at 25 °C**.** (a) electrode itself, (b) the pseudo-hybridization of DNA probe, 3'Z-probe or 5'Z-probe, (c) the hybridization of DNA probe, 3' Z-probe or 5'Z-probe and mutant type DNA target. **(D)** Histograms representing the average R_{ct} values obtained by (a) electrode itself, the pseudo-hybridization of 2.0 μg mL^{-1} (b) DNA probe, (d) 3'Z-probe or (f) 5'Z-probe, the hybridization between 2.0 μg mL^{-1} (c) DNA probe, (e) 3'Z-probe or (g) 5'Z-probe and 10.0 μg mL^{-1} mutant type DNA target.

The investigation of interaction between spermine and mutant type DNA target:

Next, the interaction of spermine and mutant type DNA target was investigated. The interaction of 2.0 μg mL^{-1} spermine and 10.0 μg mL^{-1} (equals to 1.4 μM) mutant type DNA target was performed and the impedimetric measurements were done. After the interaction of spermine and mutant type DNA target, the R_{ct} value was recorded as 1205.0 Ω which was 2.5 fold higher than the one obtained by the immobilization of the spermine. This increase was quite lower than the one obtained after the hybridization of 2.0 μg mL^{-1} Z-probe and 10.0 μg mL^{-1} mutant type DNA target (i.e. 5.7 fold) (Figure S2). It could be clearly seen that spermine had any interferrence effect onto the hybridization process.

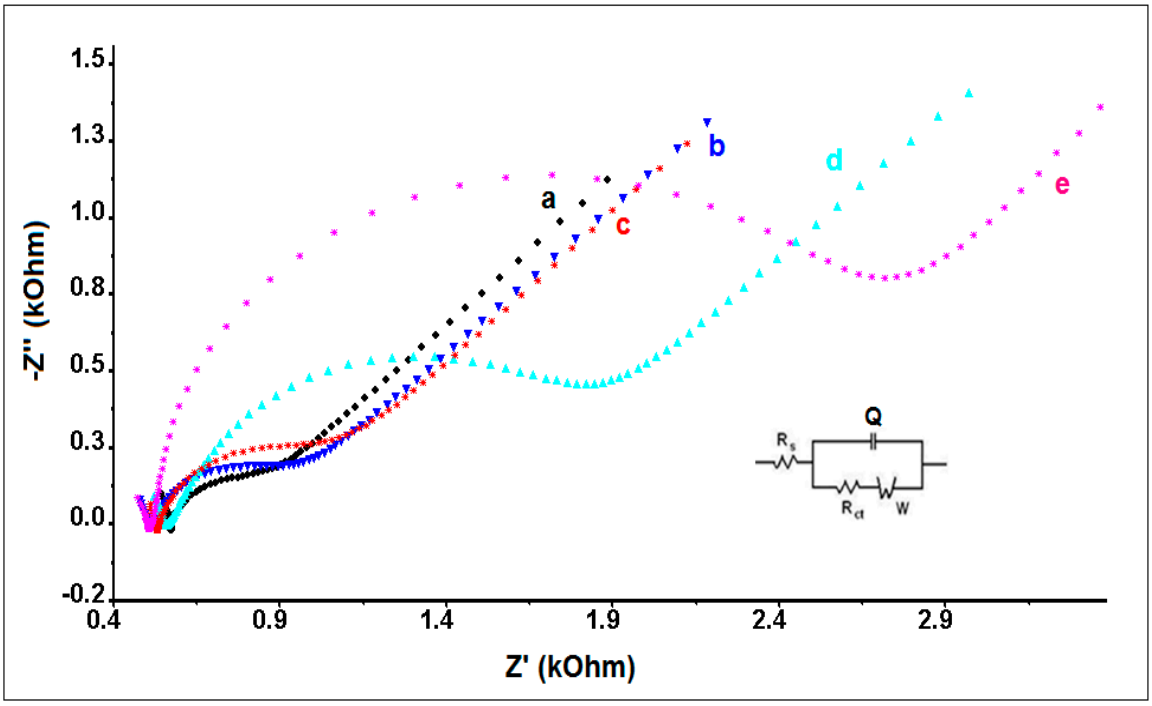

Figure S2. Nyquist diagrams obtained by **(a)** electrode itself, the immobilization of 2.0 µg mL^{-1} of **(b)** spermine or **(c)** Z-probe, and the interaction/hybridization of 2.0 µg mL^{-1} of **(d)** spermine or **(e)** Z-probe with 10.0 µg mL^{-1} mutant type DNA target.

The effect of temperature at hybridization process occurred between Z-probe and mutant type DNA target

The hybridization of 2.0 µg mL^{-1} Z-probe with 10.0 µg mL^{-1} (equals to 1.4 µM) mutant type DNA target was performed at 25 °C, 50 °C and 75 °C. After the pseudo hybridization of Z-probe at 25 °C, 50 °C and 75 °C, the R_{ct} value was measured as 386.3 Ω, 930.0 Ω and 1243.0 Ω, respectively (Figure S3). After the hybridization of Z-probe with mutant type DNA target at each hybridization temperature, an icrease at R_{ct} value about 5.7 fold, 1.7 fold and 1.8 fold was recorded. The highest increase at R_{ct} value was obtained after the hybridization occured at 25 °C and measured as 2207.4 ± 214.5 (% RSD= 9.7%, n=5). Thus, 25 ^{o}C was chosen as optimum temperature for hybridization in our study.

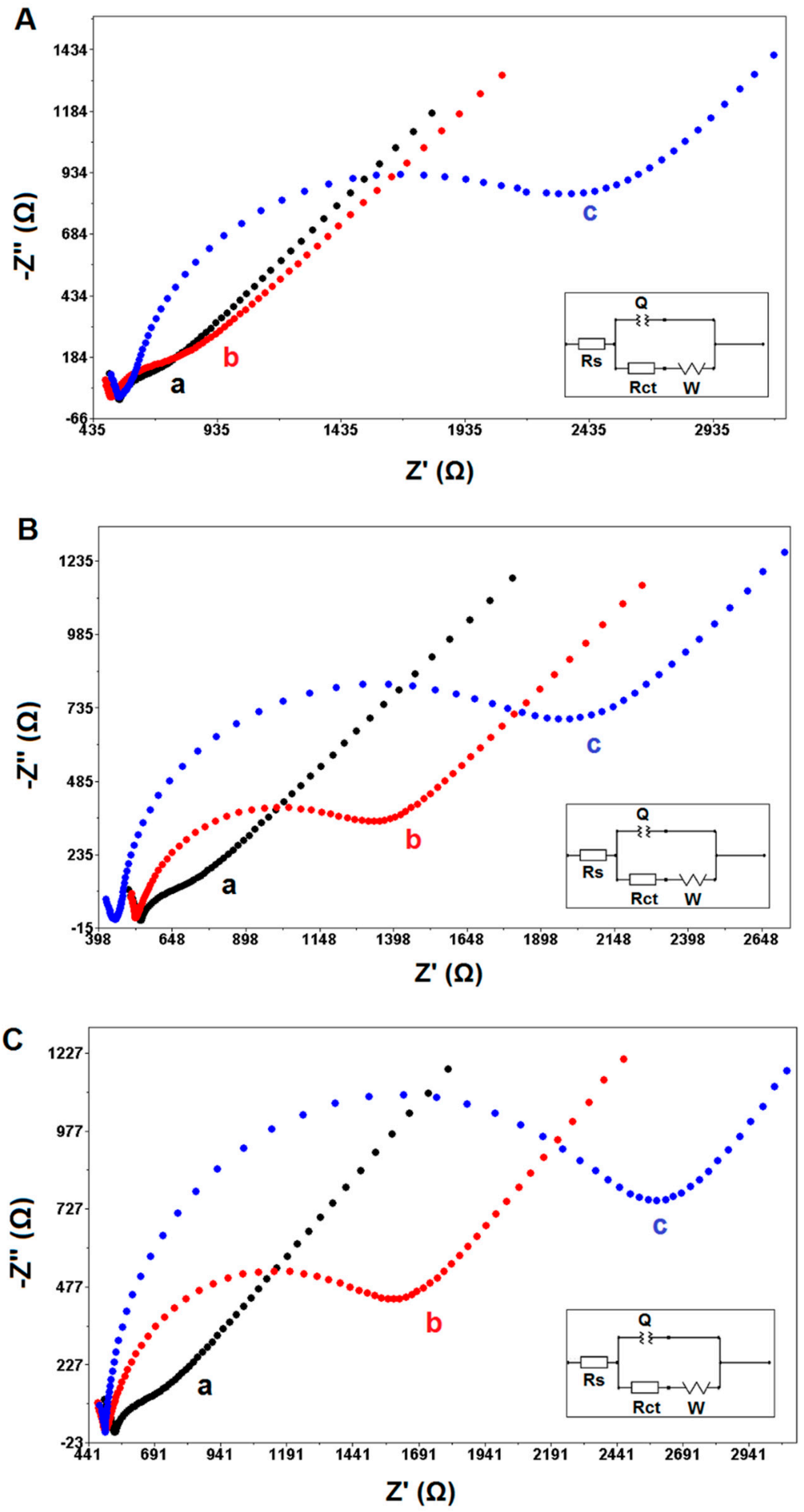

Figure S3. The Nyquist diagrams obtained after the hybridization of 2.0 µg mL^{-1} Z-probe and 10.0 µg mL^{-1} mutant type DNA target at **(A)** 25 °C**,** **(B)** 50 °C and **(C)** 75 °C. **(a)** electrode itself, **(b)** the pseudo-hybridization of Z-probe at 25 °C, 50 °C and 75 °C, **(c)** the hybridization of Z-probe and mutant type DNA target at 25 °C**,** 50 °C and 75 °C.

The effect of pH at hybridization process

The effect of pH upon the hybridization process was evaluated and the results were shown in Figure S4. The hybridization of Z-probe with mutant type DNA target was performed in different solutions, as ABS (pH 4.8), PBS (pH 7.4) or CBS (pH 9.5) and impedimetric measurements were done. After the hybridization of Z-probe and mutant type DNA target in ABS (pH 4.8), PBS (pH 7.4) or CBS (pH 9.5), the R_{ct} values were obtained as 1422.0 Ω, 2207.0 Ω and 254.0 Ω which were 3.1 fold and 5.7 fold increased than the ones obtained after the pseudo- hybridization of Z-probe in the ABS (pH 4.8) or PBS (pH 7.4) solutions. There was no change at the R_{ct} value after the hybridization in CBS (pH 9.5). However, the highest increase at the R_{ct} value was obtained in PBS (pH 7.4).Thus, it was concluded that the hybridization could be occured efficiently in the medium of PBS (pH 7.4).

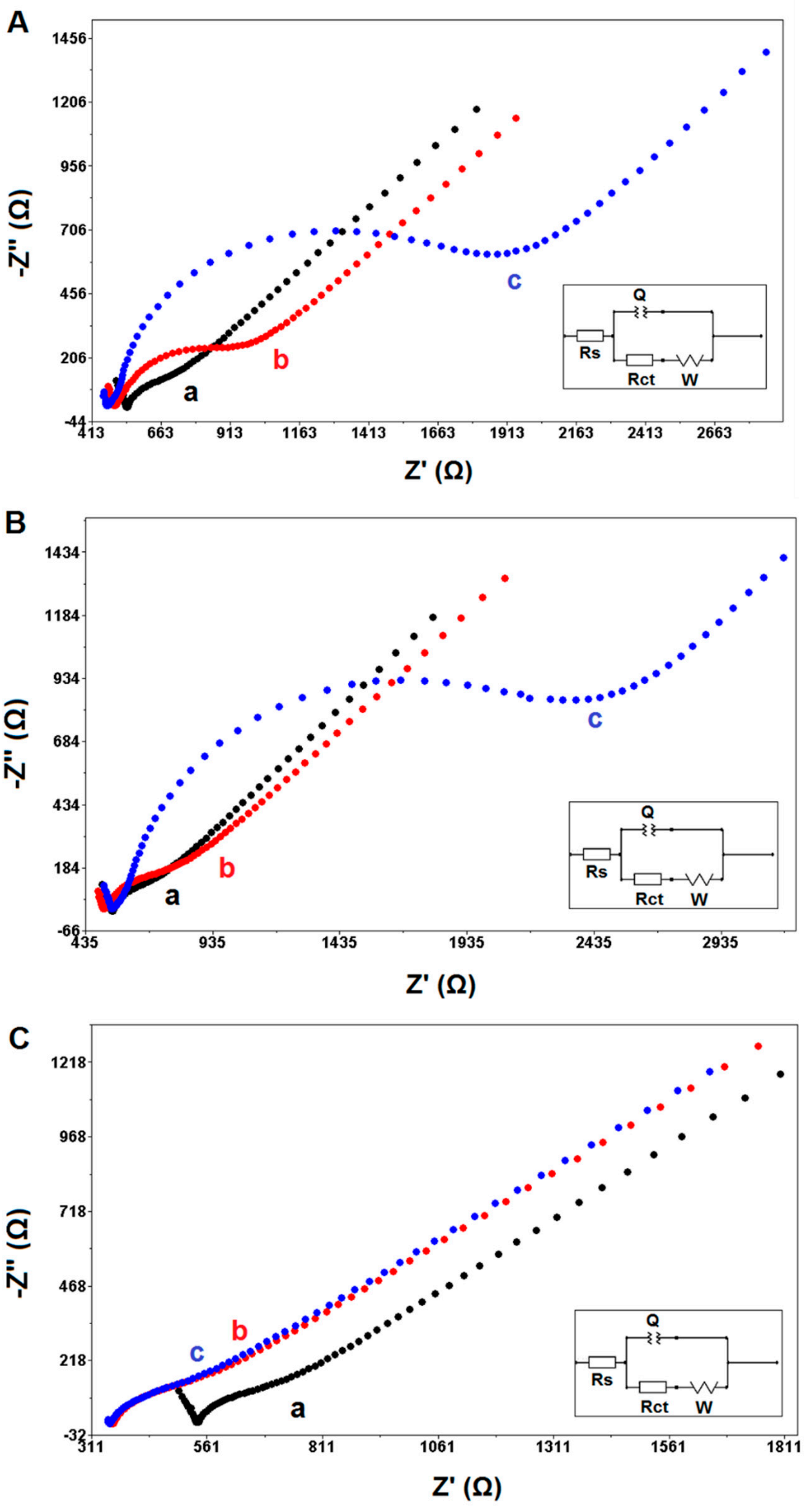

Figure S4. The Nyquist diagrams obtained after the hybridization of 2 μg mL^{-1} Z-probe and 10.0 μg mL^{-1} mutant type DNA target in **(A)** ABS (pH 4.8), **(B)** PBS (pH 7.4) and **(C)** CBS (pH 9.5). **(a)** electrode itself, **(b)** the pseudo-hybridization of Z-probe in ABS (pH 4.8), PBS (pH 7.4) or CBS (pH 9.5). **(c)** the hybridization of Z-probe and mutant type DNA target in ABS (pH 4.8), PBS (pH 7.4) or CBS (pH 9.5). Inset was the equivalent circuit model used for fitting of the impedance datas.

The effect of Mg^{2+} concentration at hybridization process

The effect of $MgCl_2$ concentration upon the hybridization was evaluated and the results were given in Figure S5. The hybridization between 2.0 µg mL^{-1} Z-probe and 10.0 µg mL^{-1} (equals to 1.4 µM) mutant type DNA target was performed in the solution of PBS (pH 7.40) or PBS containing Mg^{2+} in different concentrations as 0.5 mM and 1.0 mM. The pseudo-hybridization of Z-probe was also done in these solutions and measured as 386.3 Ω, 751.0 Ω and 645.0 Ω, respectively. After the hybridization of Z-probe and mutant type DNA target in PBS (pH 7.4) or PBS containing 0.5 mM and 1.0 mM Mg^{2+} (pH 7.4), the R_{ct} values were obtained as 2207.0 Ω, 2296.0 Ω and 2307.0 Ω which were 5.7 fold, 3.1 fold and 3.6 fold higher than the ones obtained after the pseudo-hybridization of Z-probe in this solutions. Hence, the highest increase at the R_{ct} value was obtained in PBS (pH 7.4) without any Mg^{2+}.

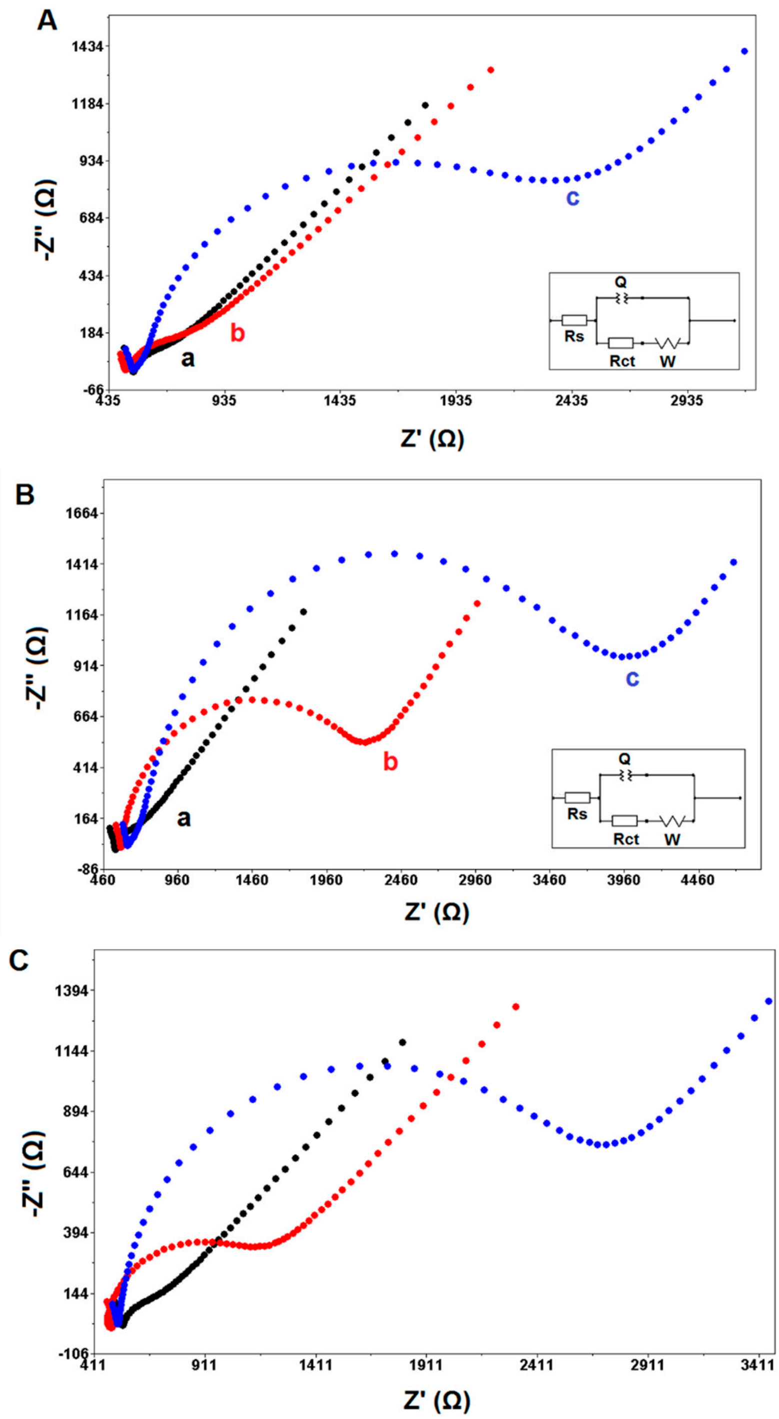

Figure S5. The Nyquist diagrams obtained after the hybridization of 2.0 μg mL^{-1} Z-probe and 10.0 μg mL^{-1} mutant type DNA target in **(A)** PBS (pH 7.4) or **(B)** 0.5 mM and **(C)** 1.0 mM Mg^{2+} contained PBS (pH 7.4). **(a)** electrode itself, **(b)** the pseudo-hybridization of Z-probe in PBS (pH 7.4), or PBS (pH 7.4) containing 0.5 mM or 1.0 mM Mg^{2+} . **(c)** the hybridization of Z-probe and mutant type DNA target in PBS (pH 7.4), or PBS (pH 7.4) containing 0.5 mM or 1.0 mM Mg^{2+}.

The effect of hybridization time at hybridization process

The hybridization between 2.0 μg mL^{-1} Z-probe and 10.0 μg mL^{-1} (equals to 1.4 μM) mutant type DNA target was performed during 5, 10 and 15 min and the changes at the R_{ct} values were evaluated in

terms of hybridization. After the hybridization of Z-probe with mutant type DNA target during 5 min, 10 min or 15 min, the R_{ct} values were measured as 2125.0 Ω, 2207.4 Ω and 2522.0 Ω which were 2.6 fold, 5.7 fold and 4.7 fold higher than the ones obtained after the pseudo hybridization of Z-probe, respectively (Figure S6). The highest increase at the R_{ct} value was obtained using 10 min hybridization time.

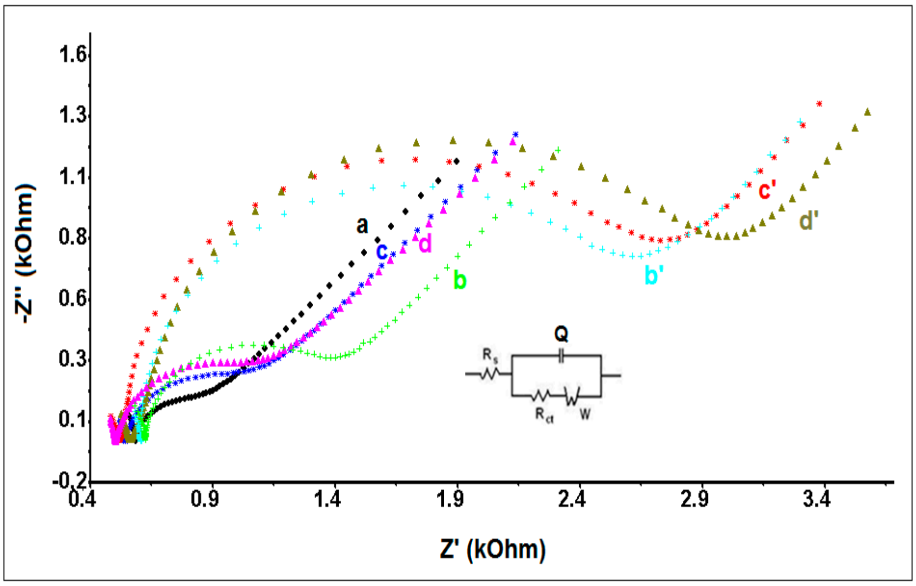

Figure S6. The Nyquist diagrams obtained after the hybridization of 2.0 μg mL^{-1} Z-probe and 10.0 μg mL^{-1} mutant type DNA target during 5 min, 10 min and 15 min. **(a)** electrode itself, the pseudo-hybridization of Z-probe during **(b)** 5 min, **(c)** 10 min, **(d)** 15min, the hybridization of Z-probe and mutant type DNA target during **(b')** 5 min, **(c')** 10 min and **(d')** 15 min.

The effect of Z- probe concentration at hybridization process:

In order to find optimum concentration of probe, the hybridization of Z-probe with 10.0 μg mL^{-1} mutant type DNA target in various concentrations of Z-probe (0.25, 0.5, 1.0, 2.0 and 4.0 μg mL^{-1}) and the changes at the R_{ct} value was measured. The highest increase at the R_{ct} value was obtained after the hybridization of 1.0 μg mL^{-1} Z-probe with 10.0 μg mL^{-1} mutant type DNA target (i.e; 2216.7 ± 233.4 Ω with the RSD%, 10.5%, n=3) (shown in Figure S7). Therefore, 1 μg mL^{-1} was chosen as optimum Z-probe concentration for our further studies.

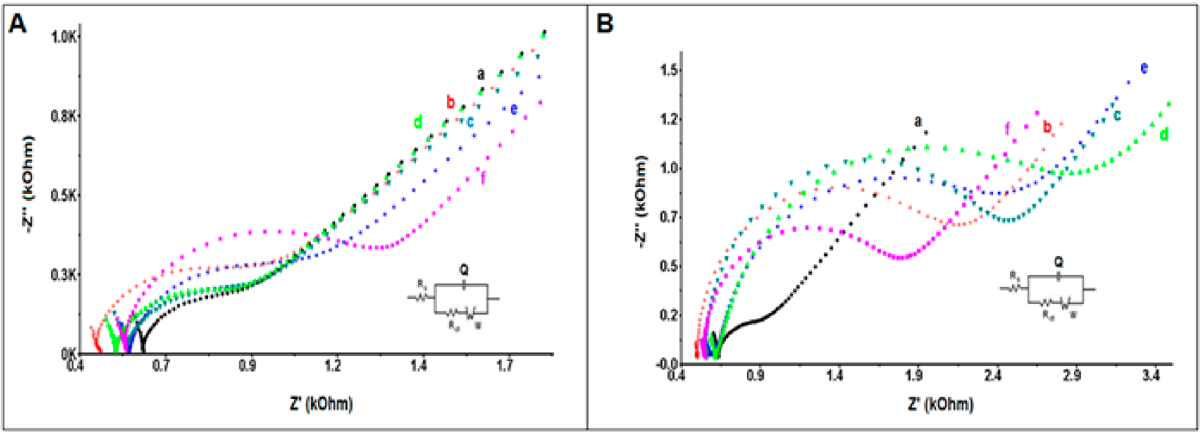

Figure S7. Nyquist diagrams of **(a)** electrode itself, before **(A)** and after **(B)** the hybridization of **(b)** 0.25, **(c)** 0.5, **(d)** 1.0, **(e)** 2.0 and **(f)** 4.0 μg mL^{-1} Z-probe and 10.0 μg mL^{-1} mutant type DNA target.

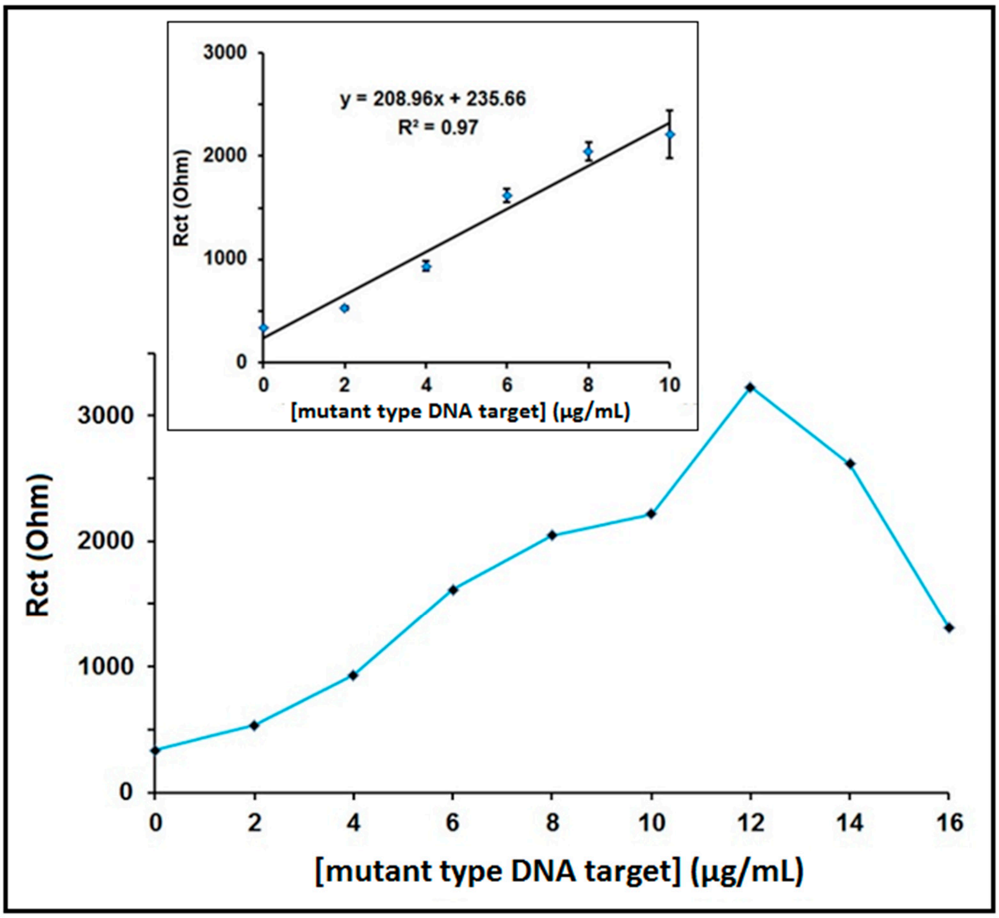

Figure S8. Line graph representing the R_{ct} values recorded by the hybridization of 1.0 μg mL^{-1} Z-probe and mutant type DNA target at the concentration level from 2.0 to 16.0 μg/mL. Inset: Calibration graph based on the average R_{ct} values (n=3) obtained after the hybridization of Z-probe with mutant type DNA target in the concentration range from 2.0 to 10.0 μg mL^{-1}.

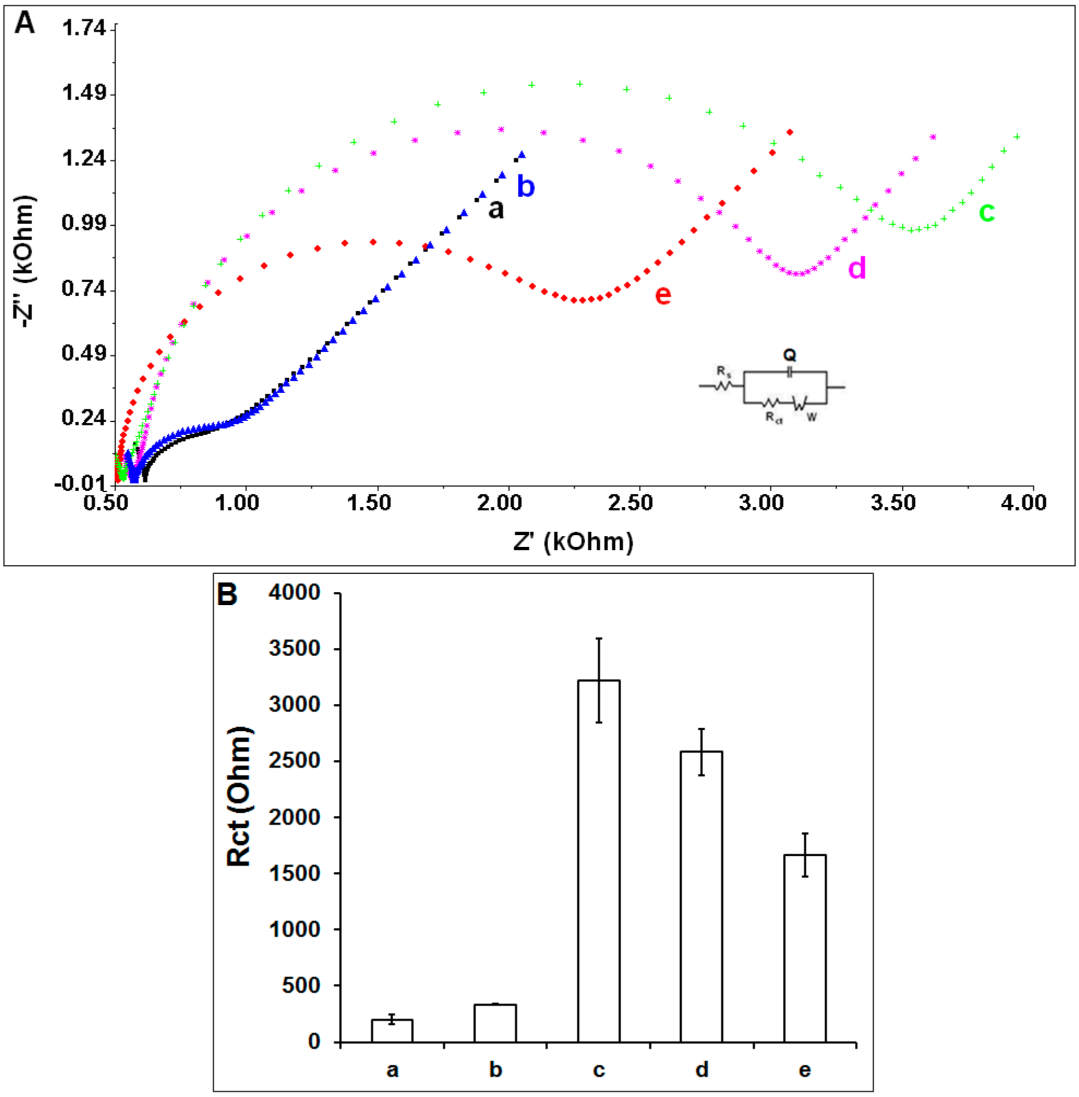

Figure S9. The hybridization of 1.0 µg mL^{-1} Z-probe and 12.0 µg mL^{-1} mutant type DNA target or C-mutant type DNA or T-mutant type DNA. **(A)** Nyquist diagrams, **(B)** histograms representing the R_{ct} values obtained by **(a)** electrode itself, **(b)** the pseudo-hybridization of Z-probe, after the hybridization of Z-probe and **(c)** mutant type DNA target, **(d)** C-mutant type DNA, **(e)** T-mutant type DNA.

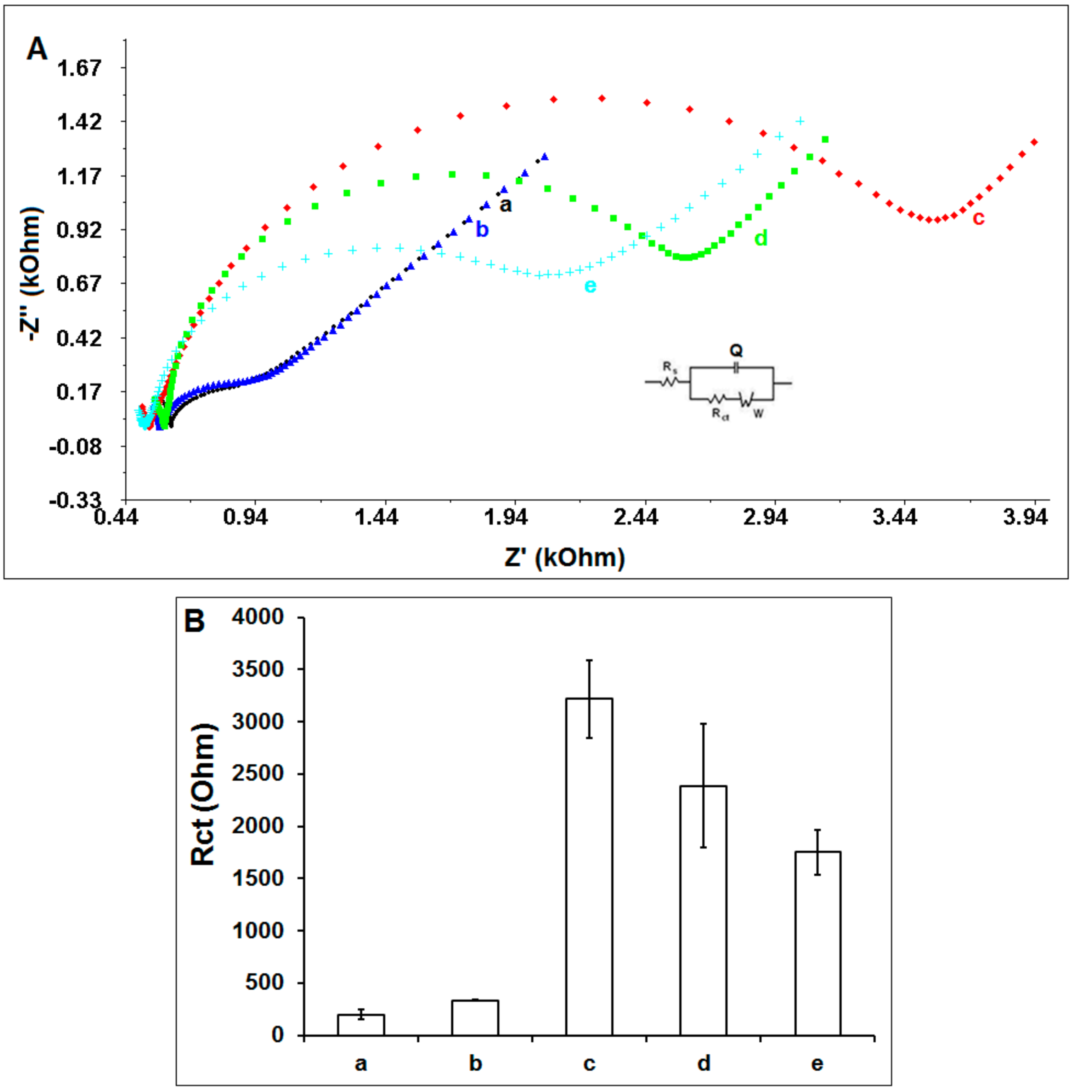

Figure S10. The hybridization of Z-probe and mutant type DNA target or ODN-1 or ODN-2. **(A)** Nyquist diagrams, **(B)** histograms representing the R_{ct} values obtained by **(a)** electrode itself, **(b)** presudo-hybridization of Z-probe, after the hybridization of Z-probe and **(c)** mutant type DNA target, **(d)** ODN-1, **(e)** ODN-2 (n=3).

Table S1. H_{Eff}% calculated based on the average R_{ct} value obtained after the hybridization of Z-probe with mutant type DNA target/ C- mutant type DNA/ T- mutant type DNA /ODN-1 / ODN-2 in contrast to the average R_{ct} value obtained in the presence of pseudo hybridization.

	**R_{ct} (Ω)**	**H_{Eff}%**
**Z-probe**	369.4 ± 59.9	-
**Z-probe and mutant type DNA target**	3219.0 ± 373.0	89.0
**Z-probe and C- mutant type DNA**	2581.5 ± 207.2	85.0
**Z-probe and T- mutant type DNA**	1664.5 ± 193.0	77.0
**Z-probe and ODN-1**	2387.0 ± 592.6	84.0
**Z-probe and ODN-2**	1751.5 ± 211.4	78.0
